# Improvement of erectile dysfunction using endothelial progenitor cells from fetal cerebral vasculature in the cavernous nerve injury of rats

**DOI:** 10.1186/s12610-022-00171-x

**Published:** 2022-12-01

**Authors:** Jae Heon Kim, Sang Hong Bak, Hee Jo Yang, Seung Whan Doo, Do Kyung Kim, Won Jae Yang, Seung U. Kim, Hong J. Lee, Yun Seob Song

**Affiliations:** 1 Department of Urology, Soonchunhyang University School of Medicine, 04401 Seoul, Republic of Korea; 2 Research Institute, e-Biogen Inc., Seoul, Republic of Korea; 3 Department of Urology, Soonchunhyang University School of Medicine, Cheonan, Republic of Korea; 4 Division of Neurology, Department of Medicine, UBC Hospital, University of British Columbia, Vancouver, Canada; 5 Medical Research Institute, Chungbuk National University, Cheongju, Chungbuk Republic of Korea

**Keywords:** Erectile dysfunction, Human endothelial cells, Human telomerase reverse transcriptase

## Abstract

**Background:**

Because of limited differentiation to endothelium from mesenchymal stem cells, it has been strongly recommended to use endothelial progenitor cells for the regeneration of the damaged endothelium of corpora cavernosa. This study was performed to investigate the immortalized human cerebral endothelial cells and their capability for repairing erectile dysfunction in a rat model of cavernous nerve injury. Circulating endothelial progenitor cells were isolated from human fetal brain vasculature at the periventricular region of telencephalic tissues. Over 95% of CD 31-positive cells were sorted and cultured for 10 days. Human cerebral endothelial progenitor cells were injected into the cavernosa of rats with cavernous nerve injury. Erectile response was then assessed. In *in vivo* assays, rats were divided into three groups: group 1, sham operation: group 2, bilateral cavernous nerve injury: and group 3, treatment with human cerebral endothelial cells after cavernous nerve injury.

**Results:**

Established immortalized circulating endothelial progenitor cells showed expression of human telomerase reverse transcriptase transcript by RT-PCR. They also showed the expression of vascular endothelial growth factor, von Willebrand factor, vascular endothelial growth factor receptor, and CD31, cell type-specific markers for endothelial cells by RT-PCR. In *in vitro* angiogenesis assays, they demonstrated tube formation that suggested morphological properties of endothelial progenitor cells. In *in vivo* assays, impaired erectile function of rat with cavernous nerve injury recovered at 2, 4, and 12 weeks after transplantation of human cerebral endothelial cells into the cavernosa.

**Conclusions:**

Telomerase reverse transcriptase-circulating endothelial progenitor cells from fetal brain vasculature could repair erectile dysfunction of rats with cavernous nerve injury.

## Résumé

**Contexte:**

En raison de la différenciation limitée de l’endothélium à partir de cellules souches mésenchymateuses, il a été fortement recommandé d’utiliser des cellules progénitrices endothéliales pour la régénération de l’endothélium endommagé des corps caverneux. Cette étude a été réalisée pour étudier les cellules endothéliales cérébrales humaines immortalisées, et leur capacité à réparer la dysfonction érectile dans un modèle de rat avec lésion du nerf caverneux. Les cellules progénitrices endothéliales circulantes ont été isolées du système vasculaire cérébral fœtal humain dans la région périventriculaire des tissus télencéphaliques. Plus de 95% des cellules CD31 positives ont été sélectionnées et cultivées pendant 10 jours. Des cellules progénitrices endothéliales cérébrales humaines ont été injectées dans les corps caverneux de rats présentant une lésion nerveuse des corps caverneux. La réponse érectile a ensuite été évaluée. Dans les essais *in vivo,* les rats ont été divisés en trois groupes: groupe 1, opération simulée; groupe 2, lésion bilatérale du nerf caverneux; et groupe 3, traitement par cellules endothéliales cérébrales humaines après lésion du nerf caverneux.

**Résultats:**

Les cellules progénitrices endothéliales circulantes immortalisées établies ont montré l’expression de la transcription de la transcriptase inverse de la télomérase humaine par RT-PCR. Elles ont également montré l’expression du facteur de croissance de l’endothélium vasculaire, du facteur de von Willebrand, du récepteur du facteur de croissance de l’endothélium vasculaire, et de CD31, marqueurs spécifiques du type cellulaire par RT-PCR pour les cellules endothéliales. Dans les essais *in vivo*, la fonction érectile altérée des rats avec lésion du nerf caverneux s’est rétablie à 2, 4 et 12 semaines après transplantation de cellules endothéliales cérébrales humaines dans les corps caverneux.

**Conclusions:**

Les cellules progénitrices endothéliales circulantes exprimant la transcriptase inverse de la télomérase, provenant du système vasculaire cérébral fœtal humain, pourraient réparer la dysfonction érectile de rats atteints de lésions des nerfs caverneux.

**Mots-clés:**

Dysfonction érectile; Cellules endothéliales humaines; Transcriptase inverse de la Télomérase humaine.

## Background

Erectile dysfunction (ED) is a common disease affecting many age groups. ED is largely due to hormonal, neurological, and vascular causes. It mainly occurs from damage to the cavernous endothelial cell (EC) or nerve damage innervating the cavernous body of the penis. It has been proposed that these cells can be repaired through transplantation of mesenchymal stem cells (MSCs) [1]. However, MSCs cannot effectively differentiate into endothelial cells. Therefore, endothelial progenitor cells (EPCs) have been strongly recommended for the regeneration of damaged endothelium of corpora cavernosa.

Although regeneration of the damaged cavernous nerve has been generated from either exogenously transplanted MSCs or transplant-mediated solicitation of endogenous stem cells, the regeneration of vasculature of the neurovascular unit is also important. Human cerebral endothelial cell transplantation into the cerebral ischemia can decrease neuroinflammation or apoptosis [2, 3].

Human telomerase reverse transcriptase (hTERT) immortalized human cerebral endothelial cells can exert therapeutic effects on experimental stroke possibly by modulating inflammation-plagued vasculome [4]. When hTERT immortalized human cerebral endothelial cells are transplanted into animals with experimental stroke, behavioral and histological deficits are attenuated accompanied by robust vasculogenesis and neurogenesis, suggesting that targeting vascular repair can activate a regenerative process [5].

The important thing in treatment using these extrapolated tissues is that cells proliferate and maintain their properties. Creation of new cultures from explanted tissue is often necessary because primary cells will age following several population doublings. Immortalized cells, which are primary cells capable of long-lasting replication, can be used to ensure a constant supply of materials through the course of our experimental work. Besides prolonging proliferation, optimal immortalized cells also display a comparable or the same genotype and phenotype as do the parent tissue. The expression of the telomerase reverse transcriptase protein (TERT) is the latest method used to achieve cell immortalization, especially in the case of cells with the highest susceptibility to telomere shortening, including human cells [6–8].

In most somatic cells, the hTERT protein is inactive. However, under conditions of exogenous expression of hTERT, cells can circumvent replicative aging by retaining enough telomere length. Investigation of many telomerase immortalized cell lines has confirmed that the use of excessive hTERT expressions to achieve cell immortalization ensures that these cells retain a stable genotype and key markers of a phenotype. Earlier research has successfully used hTERT to immortalize human cerebral endothelial progenitor cells [4, 5]. To objective of the present study was to establish human telomerase reverse transcriptase immortalized cerebral endothelial progenitor cells (HEN6) and to examine its capability to repair erectile dysfunction with cavernous nerve injury (CNI).

## Materials and methods

### Preparation of human cerebral endothelial cells

This study was conducted using our previous research method [5].

HEN6 cells were prepared from human fetal cerebral vasculature. The Institutional Animal Care and Use Committee of the hospital granted approval for study procedures, which were carried out in line with the guidelines of the National Institute of Health Guide for the Care and Use of Laboratory Animals. The periventricular area of telencephalic tissues of 11 to 14 weeks human fetal cadavers was used. We initially established primary cell cultures from the cerebral vasculature of these human fetuses [5].

After careful separation from the brain, the fetal cerebral vasculature was dissected into small fragments and then subjected them to homogenization with four strokes of a 1 mL Teflon-glass homogenizer (Wheaton, Millville, NJ, USA). Subsequently, the tissue debris was subjected to centrifugation at 600 g for five minutes at 4 °C. The next step was resuspension of the homogenate in 1 mL of 0.1% Collagenase/Dispase® (Boehringer Mannheim, Mannheim, Germany) in Ca2- and Mg2-free HBSS (Hanks Balanced Salt Solution) with 100 U/mL penicillin, 100 mg mL streptomycin, 20 U/mL DNase I, and 0.147 mg/mL Tosyl-lysine-chloromethyl ketone at pH 7.4. This mixture was incubated in a water bath at 37 °C for 60 min with agitation. The mixture was then centrifuged at 600 g for five minutes at 4 °C. The pellet was resuspended in 10 mL 25% BSA (bovine serum albumin) in HBSS and centrifuged at 1000 g for 15 min at 4 °C. The pellet was then resuspended in 1 mL of 0.1% collagenase/Dispase solution as previously outlined. After 10 min of incubation at 37 °C with sporadic agitation, it was again centrifuged at 1000 g for 15 min at 4 °C, yielding a pellet that contained a fraction abundant with retinal capillaries.

For seeding the fraction abundant with capillaries, tissue culture dishes (Becton Dickinson, Bedford, MA, USA) with a coating of rat tail type I collagen were used. Cells were cultured in DMEM (Dulbecco’s modified Eagle’s medium) with a composition of 20 mm of sodium bicarbonate, 15 ng/mL of ECGF (endothelial cell growth factor), 10 U/mL of heparin, 100 U/mL of penicillin, 100 mg/mL of streptomycin, 2.5 mg/mL of amphotericin B, and 20% FBS (fetal bovine serum). Culture conditions were: temperature of 37 °C, humidified atmosphere, and 5% carbon dioxide/air. A variety of colony types were distinguished after 14 days. The colony with a spindle-fiber morphology was enveloped with a stainless-steel penicillin cup and subjected to selective trypsinization. Meanwhile, colonies of pericytes, fibroblasts and other types of cells were eliminated via aspiration. After they underwent passage two to three times, cells underwent cloning from one cell via colony formation and double separation from other cells with a penicillin cup.

Once migration of circulating endothelial progenitor cell (CEPC), explants were eliminated. Colonies were monitored to keep track of contamination of smooth muscle cells. CEPC proliferation was permitted. A cell sorter (FACS Vantage, Becton Dickinson, Bedford, MA, USA) with fluorescence activation to classify cells with PE-conjugated anti-CD31 antibody (1:50; Becton Dickinson, Bedford, MA, USA), yielding more than 95% CD31-positive cells. Cells were validated to have an endothelial source via assimilation of Alexa-488-acetylated-LDL (Molecular Probes, Eugene, OR, USA), immunofluorescence for CD-31 (Becton Dickinson, Bedford, MA, USA), Vascular Endothelial Growth Factors (VEGF), von-Willebrand factor (vWF) (Abcam, Cambridge, UK), and vascular endothelial growth factor receptor2 (KDR) (Sigma Co., St Louis, MO, USA).

### Immortalized human endothelial progenitor cells

Transduction of telomerase into CEPC was performed. Previously characterized primary dissociated telencephalic cells were subjected to replication-incompetence amphotropic infection with a retroviral vector underpinned by a Maloney murine leukemia viral (MMLV) backbone. A tetracycline (Tet)-response regulator vector was initially inserted into the culture. Resistance to G418 (300 μg/mL) was the basis for infectant selection. Subsequently, a telomerase-encoding retroviral vector was introduced into chosen clones. Infectant selection was based on hygromycin resistance of 200 μg/mL. A significant proportion of cells died during antibiotic-based selection (resistance to G418 and to hygromycin). This led to the emergence of colonies of phase-dark bipolar or polygonal cells displaying short processes [5]

### Cell culture

Tissue culture dishes with a coating of 2% gelatin type B (Sigma Co., St Louis, MO, USA) were used for culturing hTERT immortalized HEN6. The culture environment was M199 (medium 199) (GIBCO, Waltham, MA, USA), to which 20% FBS, EGM-2MV (Clonetics), L-glutamine, 10,000 /mL penicillin, 10,000 /mL streptomycin, and 25 g/mL Fungizone were added. Growth took place in a humidified incubator at a temperature of 37 °C and 5% carbon dioxide, with a change of medium at three-day intervals [5]

### Cytogenetics

Cytogenetic analysis of hTERT-hEPCs were performed to confirm the normal human karyotype of chromosomes.

### Confirmation of HEN6

Cell lines were isolated by limiting dilution. Their clonal identity was confirmed. reverse transcription polymerase chain reaction (RT-PCR) was performed to confirm the presence of telomerase messenger ribonucleic acid (mRNA) [5]

### In vitro angiogenesis assay

We used 96-well tissue culture plates with a coating of 30 μL Matrigel (BD Biosciences, Franklin, NJ, USA) for seeding ECs, each well containing 5000–20,000 cells. After one day, we used an inverted microscope with 40 × magnification to view cells and to ascertain whether tubules resembling capillaries had formed.

### Generation of cavernous nerve injured ED model and transplantation of HEN6 into the penile cavernosum

The Institutional Animal Care and Use Committee of the researcher’s hospital granted approval for all procedures, which we performed in line with the guidelines of the National Institute of Health Guide for the Care and Use of Laboratory Animals (2001). A total of 54 Sprague–Dawley eight-week-old male rats were equally divided into three groups at 2, 4 and 12 weeks: group 1, sham operation; group 2, bilateral CNI; and group 3, treated with HEN6 (1 × 10^6^ cells) following CNI.

A solution of 30 mg/kg of 1% ketamine and 4 mg/kg xylazine hydrochloride was used to anesthetize rats. Cavernous nerve (CN) and major pelvic ganglion (MPG) posterolaterally on each side of the prostate were observed. No additional procedures in group 1. For rats in group 2 and 3, a hemostat was employed to isolate and crush the CN for two minutes on each side at a distance of 5 mm from its MPG origin. Next, a penile skin incision was made to dissect and palpate the penile cavernosum. For rats in group 3, HEN6 (1 × 10^6^ cells) was transplanted into the cavernosa [9]. To prevent infection, Flomoxef (cephalosporin; Ildong, Seoul, Korea) was injected intraperitoneally at 10 mg/kg daily. The penile cavernosum was harvested at 2, 4, and 12 weeks after cell transplantation.

### Measurement of erectile function after transplantation

After hTERT-hEPCs were transplanted, erectile responses in groups 1 to 3 were assessed at two, four, and 12 weeks after transplantation. A solution of 30 mg/kg 1% ketamine and 4 mg/kg xylazine hydrochloride was administered through an intraperitoneal injection to anesthetize rats. A midline laparotomy was subsequently performed for bilateral exposure of the main pelvic ganglia and cavernous nerve. Exposure of the penis and mobilization of the corpus spongiosum was then performed so that a 25-gauge needle could be inserted into the corpus cavernosum. A polyethylene-50 tube was used to attach a needle to a pressure transducer containing heparinized saline. After that, a bipolar electrode made of platinum was positioned around the cavernous nerve so that penile erections could be induced through electric stimulation. For electric stimulation, 1 and 5 V were used at 12-Hz frequency with a 1-ms square-wave interval for 60 s. The highest intracavernous pressure (ICP) was then calculated [10].

### Immunohistochemical study

For immunohistochemistry, he cells were placed onto glass slides and incubated with 5% goat normal serum with 1% BSA (Jackson ImmunoResearch, West Grove, PA, USA) for blocking purposes. Slides were then incubated with primary antibodies of SMA (smooth muscle actin) (1:100; Sigma Co., St Louis, MO, USA) and vWF (1:100; Sigma Co., St Louis, MO, USA) at 22 °C for half an hour without light exposure. After washing out, slides were incubated Alexa-488, and Alexa-594 conjugated goat anti-mouse or anti-rabbit secondary antibodies (1:800; Molecular Probes, Eugene, OR, USA) diluted in blocking buffer at 22 °C for half an hour without. Slides were subsequently washed and incubated with DAPI (4’,6-diamidino-2-phenylindole) (1:30,000; Molecular Probes, Eugene, OR, USA) for five minutes to achieve cell nucleus staining [10]. Cell morphology and fluorescence were observed at 488- and 594-nm excitation wave-lengths using a microscope (Olympus, Tokyo, Japan). Quantitative analysis of histologic examinations was processed with an image analyzer system (National Institutes of Health [NIH] Image J 1.34, https://imagej.nih.gov/ij/).

### Statistical analysis

Transplantation of stem cells was investigated using via two-way ANOVA (Analysis of variance) and post-hoc Tukey test. Data are expressed as mean ± SE (standard error), with statistical significance indicated by a *P*-value of less than 0.05.

## Results

### Immortalization of HEN6 and cytogenetics

**Fig. 1:**
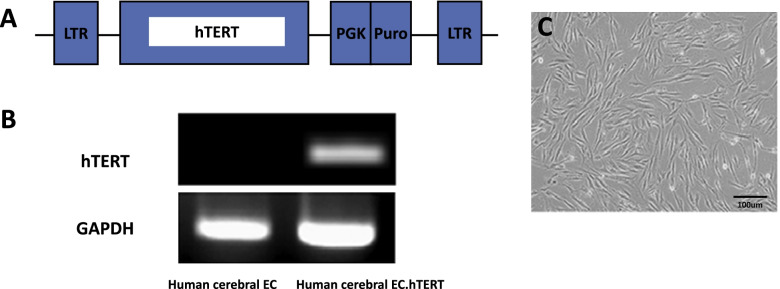
HEN6 expresses human telomerase immortalized cerebral endothelial cell. **A** HEN6 cells were infected with a retroviral vector encoding telomerase. **B** The expression of CE transcript or protein was confirmed by RT-PCR or western blot. The result of a RT-PCR analysis for the expression of the human telomerase reverse transcriptase (hTERT) protein in HEN6. **C** Phase microscopic image of HEN6 (× 40). EC = endothelial cell, HEN6 = human telomerase reverse transcriptase immortalized cerebral endothelial progenitor cells, GAPDH = Glyceraldehyde 3-phosphate dehydrogenase

**Fig. 2:**
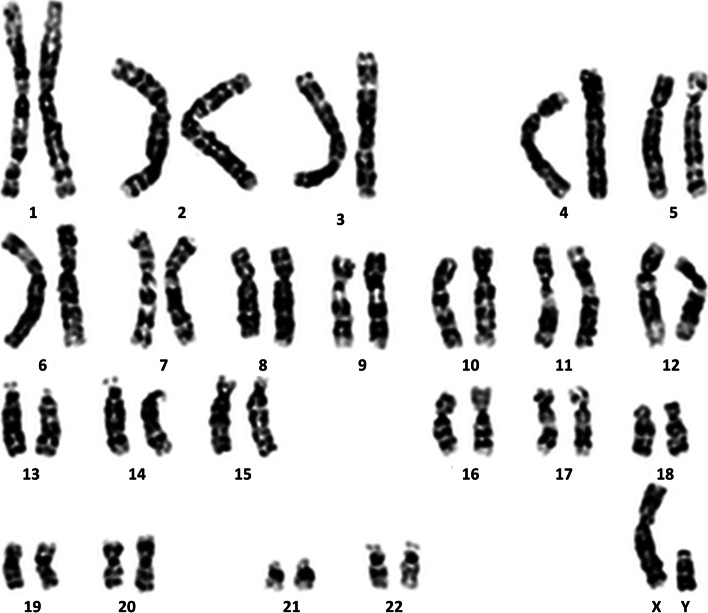
Cytogenetic analysis of HEN6. Cytogenetic analysis indicates that HEN6 carries normal human karyotype of chromosomes, 22 pairs of autosomes, and one X chromosome, one Y chromosome. HEN6 = human telomerase reverse transcriptase immortalized cerebral endothelial progenitor cells

We had established immortalized HEN6. The established immortalized hTERT-hEPCs showed expression of telomerase transcript by RT-PCR (Fig. 1). Cytogenetic analysis indicates that HEN6 carried normal human karyotype of chromosomes, 22 pairs of autosomes, one X chromosome, and one Y chromosome (Fig. 2). HEN6 demonstrated genotype and phenotype identical to those of their parental tissue.

### Flowcytometry of HEN6

**Fig. 3:**
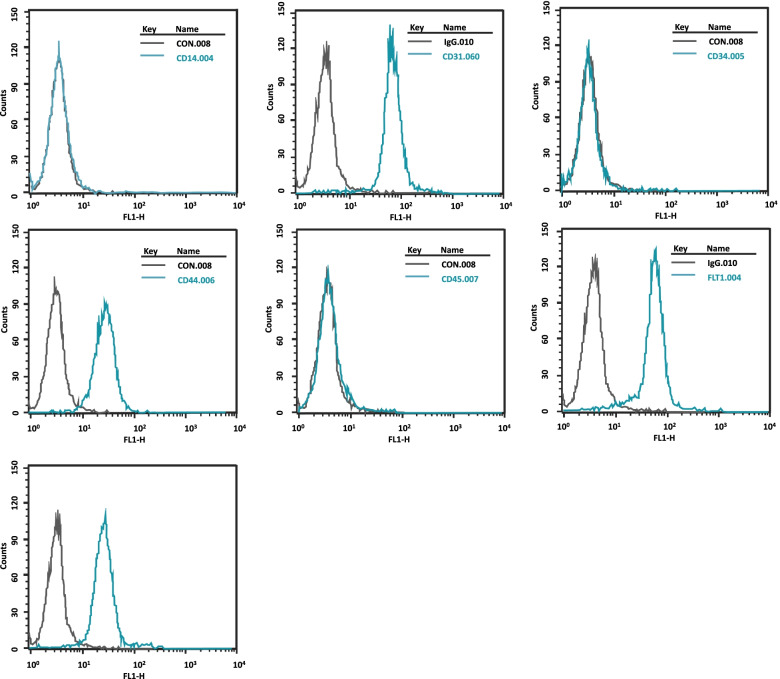
Flowcytometry of HEN6. HEN6 showed expression of endothelial marker (CD 31, CD 44, FLT1), but not hematopoietic stem cells markers CD34 or CD45. HEN6 = human telomerase reverse transcriptase immortalized cerebral endothelial progenitor cells

HEN6 showed expression of endothelial markers (CD 31, CD 44, FLT1 (vascular endothelial cell growth factor receptor 1), and KDR). However, it did not express endothelial marker CD34 or CD45 for hematopoietic stem cells (Fig. 3). This means that HEN6 has the potential of endothelial cells. And, modification of CEPC with hTERT did not alter endothelial progenitor cell properties.

### Immunohistochemical study of HEN6

**Fig. 4:**
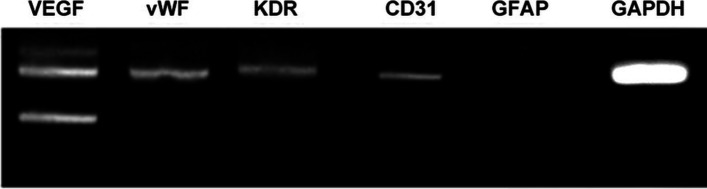
Immunohistochemical study of HEN6. HEN6 expressed endothelial markers of VEGF, vWF, KDR, CD31 without expressing neuronal marker of GFAP by RT-PCR. GFAP = glial fibrillary acidic protein. VEGF = vascular endothelial cell growth factor, KDR = vascular endothelial cell growth factor receptor 2, vWF = von Willebrand factor, HEN6 = human telomerase reverse transcriptase immortalized cerebral endothelial progenitor cells, GAPDH = Glyceraldehyde 3-phosphate dehydrogenase

RT-PCR revealed that HEN6 expressed of CD31, VEGF, vWF, and KDR (Fig. 4), known to be specific markers for hTERT-hEPCs. These cells displayed more than 99% positivity for such markers. HEN6 demonstrated tube formation, which created a complex three-dimensional capillary network, suggesting morphological properties of endothelial progenitor cells.

### Erectile response to nerve stimulation in cavernous nerve-injured model of rats

**Fig. 5:**
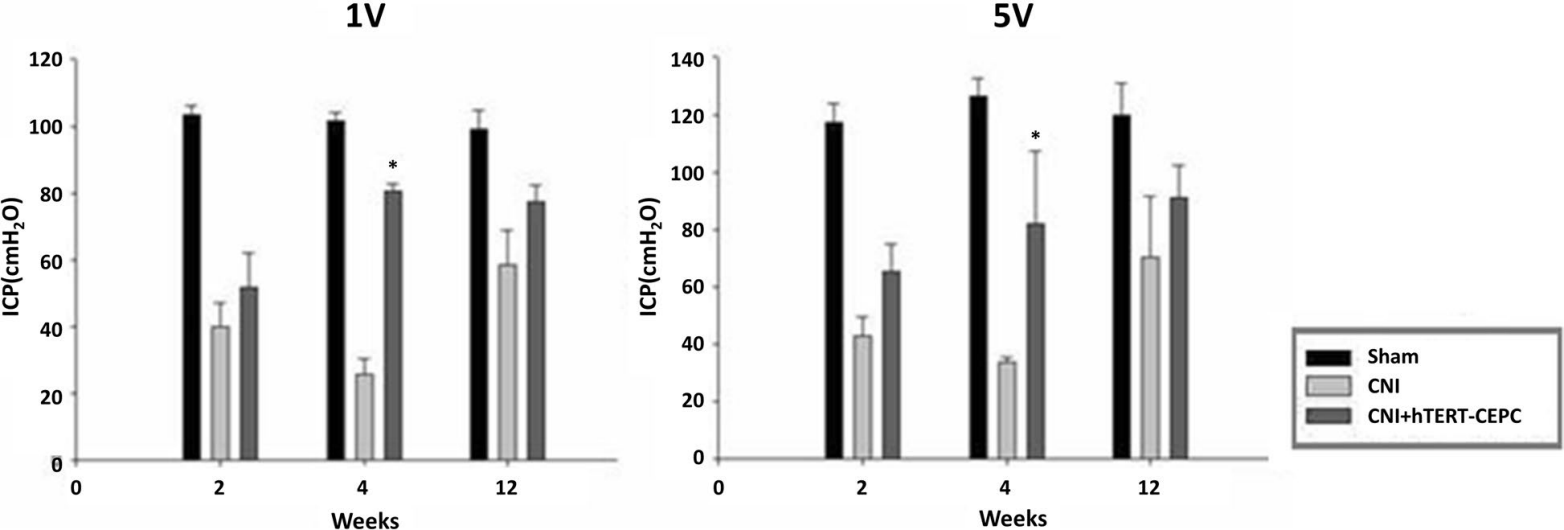
Erectile response to nerve stimulation in cavernous nerve-injured model of rats. At 2, 4, 12 weeks, the group with cavernous nerve injury showed significantly lower erectile function than the group without cavernous nerve injury (*P* < 0.05). The group transplanted with HEN6 showed higher erectile function than the group without HEN6 (*P < 0.05). For electric stimulation, 1 and 5 V were used at 12-Hz frequency with a 1-ms square-wave interval for 60 s. The number of rats used in the study was 6 for each study interval and group. ICP = intracavernous pressure, HEN6 = human telomerase reverse transcriptase immortalized cerebral endothelial progenitor cells, CNI = cavernous nerve injury without stem cells transplantation, hTERT-CEPC = Human telomerase reverse transcriptase-circulating endothelial progenitor cell

The CNI model of rats showed decreased ratio of maximal intracavernous pressure (ICP) than rats in the control group in response to cavernous nerve stimulation. ICP recovered faster in HEN6 transplanted group than in the CNI group, with the difference being the greatest at 4 weeks (*P* < 0.05). However, ICP recovered in the CNI group at 12 weeks, showing no difference from the HEN6 transplanted group (Fig. 5).

### Expression of cavernous smooth muscle and endothelial makers

**Fig. 6:**
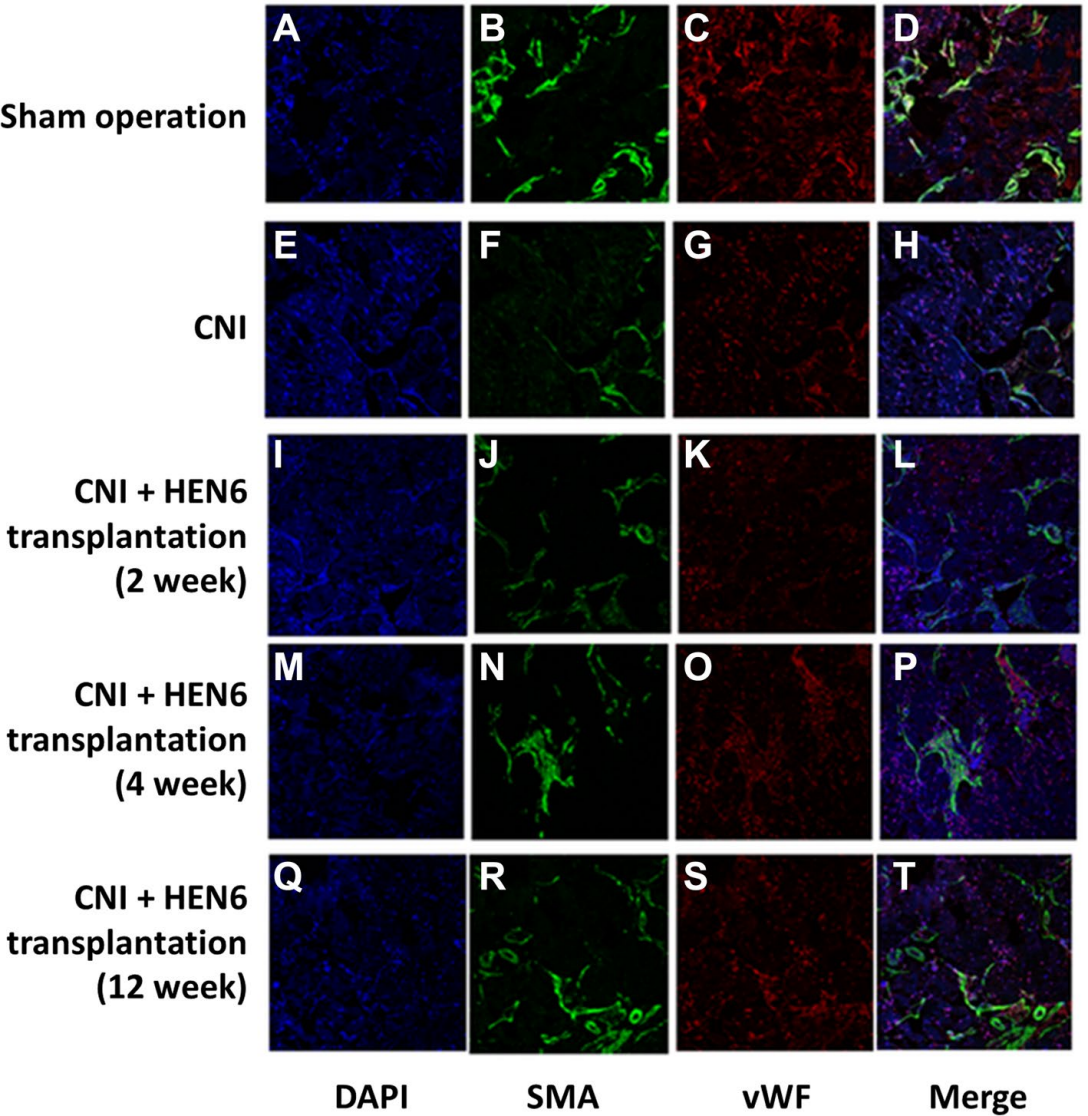
Expression of cavernous smooth muscle and endothelial makers. Expression of cavernous smooth muscle and endothelial makers after transplantation of HEN6. **A**-**D** Sham operation group, **E**–**H** Cavernous nerve injury group, **I**-**L** HEN6 transplantation group at 2 weeks after transplantation. **M**-**P** HEN6 transplantation group at 4 weeks after transplantation. **Q**-**T** HEN6 transplantation group at 12 weeks after transplantation. The group with cavernous nerve injury showed decreased density of expression of cavernous endothelial and smooth muscle makers than the sham operation group. The group with transplantation of HEN6 showed increased expression of cavernous endothelial and smooth muscle makers than the cavernous nerve injury group. Blue = DAPI, Green = SMA, Red = vWF. Sham = sham operation, CNI = cavernous nerve injury without stem cells transplantation. DAPI = 4’,6-diamidino-2-phenylindole, SMA = smooth muscle actin, vWF = von Willebrand factor, HEN6 = human telomerase reverse transcriptase immortalized cerebral endothelial progenitor cells

**Fig. 7:**
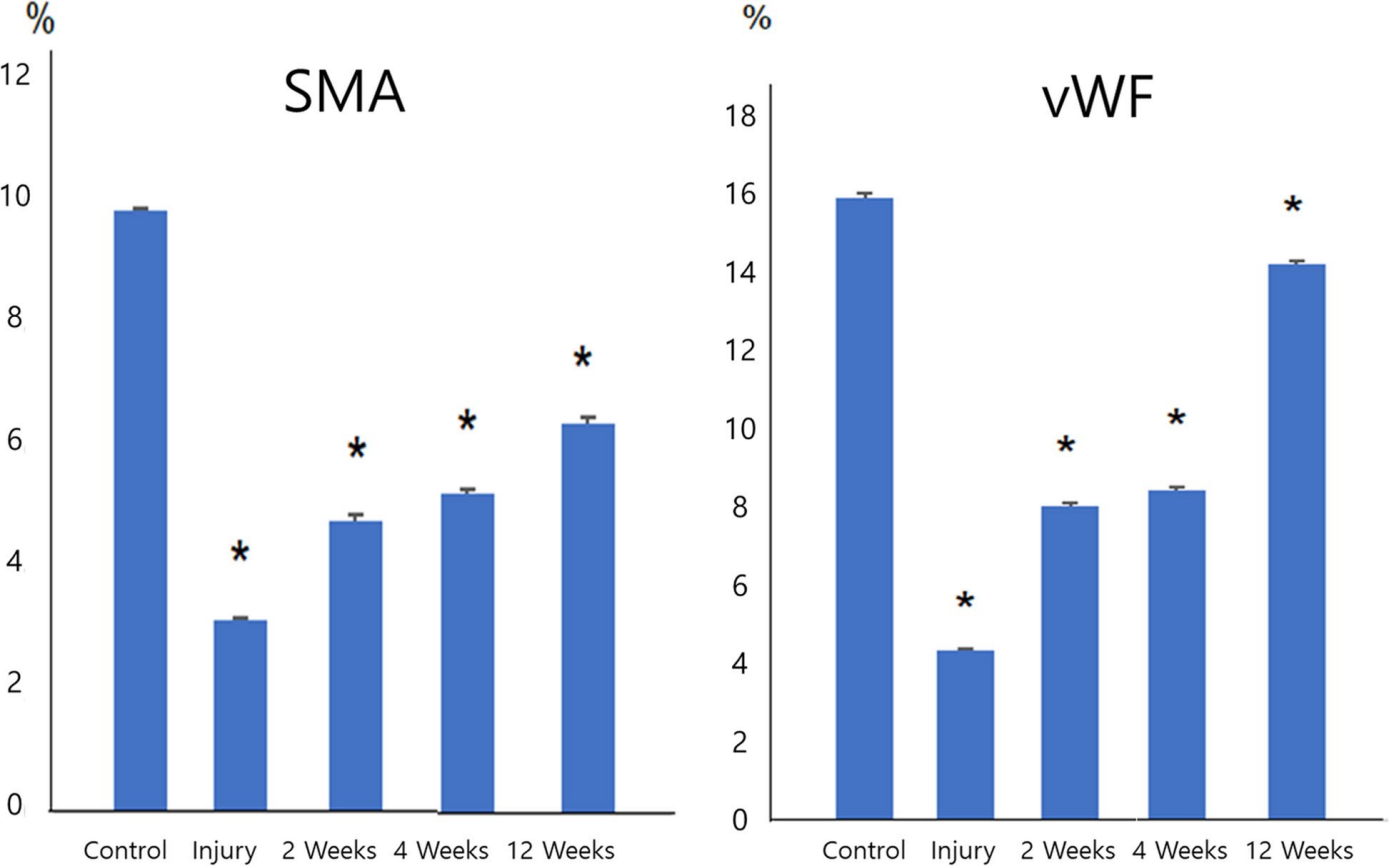
Quantitative analysis of SMA, vWF staining area. Expression levels of cavernous endothelial and smooth muscle makers were increased in the groups with transplantation of HEN6 than the cavernous nerve-injured group. The number of rats used in the study was 6 for each study interval and group. *P < 0.05 (Sham Vs CNI, CNI Vs 2 weeks, CNI Vs 4 weeks, CNI Vs 12 weeks). SMA = smooth muscle actin, vWF = von Willebrand factor, HEN6 = human telomerase reverse transcriptase immortalized cerebral endothelial progenitor cells

At 2, 4, and 12 weeks, the group with cavernous nerve injury showed decreased density of expression of cavernous endothelial and smooth muscle makers than the sham operation group. Expression levels of cavernous endothelial and smooth muscle makers were increased in the groups with transplantation of HEN6 than the cavernous nerve-injured group (Figs. 6 and 7).

## Discussion

The immortalized CEPCs established in our study expressed cell type-specific markers of ECs and showed tubular formation indicative of angiogenesis. In addition, ED treatment using immortalized CEPCs restored the impaired erectile function of CNI rat. Although there are currently available treatment options for ED, it is difficult for clinicians to treat ED. The reason is that such treatments lack effectiveness in some patients and it is not a treatment for the cause of ED. Our study to improve the effect of stem cells will be an important attempt in the treatment of ED.

Lately, a new awareness has arisen about the significance of endothelial cells for the regulation of vasculogenesis, as well as neurogenesis [11, 12]. Structural elements, neurotransmitter and ion modulations, and blood flow controls are just some of the wide range of cerebrovascular maintenance functions that cerebral endothelial cells fulfill [13, 14]. Research evidence from animal models of stroke has confirmed that transplantation of exogenous endothelial progenitor cells can be beneficial for clinical treatment [15, 16].

Damage to the cavernous nerve impairs the vascular functions that the penile cavernosum fulfills. Pathophysiological ED associated with such damage might be alleviated by devising interventions that can safeguard the vasculature against modifications that cavernous nerve damage causes.

Cultures of endothelial progenitor cells are useful because they offer an uncontaminated and uniform cell population. However, such cultures are disadvantageous because of their limited yield, even following repeated passages in culture [17, 18]. HEN6 provides a high yield of cells to permit studies that require large amounts of material. HEN6 is an immortalized human cerebral endothelial progenitor cell line containing hTERT (Fig. 1). Since CEPC cannot be obtained easily with difficulty in its storage, generation of immortalized CEPC is a good solution. As hTERT does not induce malignancy by the modification of CEPC with hTERT, immortalized HEN6 can be a vehicle for the transduction of therapeutic genes [19].

The source of CEPC was the primary dissociated cell cultures derived from the periventricular area of 14-week human fetal telencephalic tissues that had matured for ten days. We then subjected these cells to hTERT infection. After we had subcultured HEN6 at almost 90% confluence, we carried out additional experiments. In terms of immunocytochemistry, these cells displayed more than 99% positivity for several phenotypic markers, including vWF, a human mitochondrial marker, and CD31 [5]. Morphological characteristics and occurrence of distinctive endothelial cell markers (i.e., VEGF, vWF, KDR and CD31) provided the basis on which we detected isolated endothelial cells in the present study (Fig. 4).

Despite its apparent therapeutic efficacy, *in vivo* monitoring of this EPC transplanted into the penile cavernosum has rarely been investigated. To methodically scrutinize cell therapy, it is necessary to monitor transplanted cells *in vivo*. Most research studies that have attempted this employed immunohistochemical staining, which required experimental animals to be destroyed. However, one can avoid this while also achieving assessment in the same host by noninvasive monitoring of biological activities of transplanted stem cells via nanoparticle-based molecular imaging. It has been demonstrated that iron nanoparticle-labeled cells are more stable *in vivo* and permit more pronounced contrast [20–23].

Transplantation of hTERT- CEPC can ameliorate stroke-induced behavioral deficits dose dependently [4, 5]. In the present study, transplantation of HEN6 increased intracavernous pressure in erectile response of HEN6 to nerve stimulation in rats having ED with CNI (Fig. 5). This result indicates that HEN6 can lead to recovery of erectile dysfunction.

In this study, the group with transplantation of HEN6 showed increased expression of cavernous endothelial and smooth muscle makers than the CNI group (Figs. 6 and 7). It also showed vasculogenesis and enrichment of smooth muscle of cavernosum. Transplantation of EPC can induce vasculogenesis in endogenous and exogenous cells and promote endogenous neurogenesis mediated neuroprotective cellular processes [4, 5]. Inflammation-related secondary cell death of stroke could be avoided by implementing EPC therapy in vasculature-directed treatment [4, 5]. The basis of this approach is substitution of injured endothelial cells. Additionally, repair can be promoted by EPC through production of growth factors via paracrine mechanisms [24, 25]. Vasculogenesis in endogenous and exogenous cells, endogenous neurogenesis mediated neuroprotective cellular processes, and abrogation of the inflammation-associated secondary cell death might be proposed as mechanisms for therapeutic effects [4].

Our study has some limitations. In 1992, Chen et al. studied normal range of ICI using male, adult Sprague–Dawley rats (200 to 300 gm) [26]. They measured ICP and reported an ICP of 6.1 mmHg (82.9 mmH_2_O) when saline was injected. The measured ICP in our study was 90-100mmH_2_O even in the sham operation group. Usually, these measurements appear under very well-controlled conditions. We suggest that the relative lower ICP results of the CNI than those of the sham operation group are important for the exact figure of ICP for identifying ED in rats than the absolute ICP values. And, as HEN6 comes from human cerebral vasculature, they have the advantage of clinical application. However, there are no studies yet using HEN6 in human erectile dysfunction.

## Conclusion

Because of limited differentiation to endothelium from mesenchymal stem cells, EPC have been strongly recommended for the regeneration of damaged endothelium of corpora cavernosa. According to our result that transplanted HEN6 cells into the cavernosum of rats with damaged cavernous nerves, improved erectile function, we could suggest that HEN6 can be a useful tool for treating erectile dysfunction. Further studies on the therapeutic role of HEN6 in ED and application studies in humans are needed.

## Data Availability

The datasets generated during and/or analysed during the current study are available from the corresponding author on reasonable request.

## Abbreviations

CNI: Cavernous nerve injury
CEPC: Circulating endothelial progenitor cell
CD: Cluster of differentiation
EC: Endothelial cell
EPC: Endothelial progenitor cells
ED: Erectile dysfunction
HEN6: Human cerebral endothelial cells
hTERT: Human telomerase reverse transcriptase
ICP: Intracavernous pressure
KDR: Vascular endothelial growth factor receptor2)
MSC: Mesenchymal stem cells
mRNA: Messenger ribonucleic acid
MMLV: Maloney murine leukemia viral
NIH: National Institutes of Health
RT-PCR: Reverse transcription polymerase chain reaction
TERT: Telomerase reverse transcriptase protein
Tet: Tetracycline
VEGF: Vascular Endothelial Growth Factor
vWF: Von Willebrand factor A
